# Supplementary material for: BNT162b2 COVID-19 Vaccine Safety among Healthcare Workers of a Tertiary Hospital in Italy
Source: Vaccines (Basel). 2023 Feb 17;11(2):477. doi: 10.3390/vaccines11020477 (PMC9964542; doi:10.3390/vaccines11020477)
Supplement: Supplementary file 1 [file vaccines-11-00477-s001.zip › vaccines-2223195-supplementary/vaccines-2223195-sup-final/vaccines-2223195 -Tables.pdf]

Table S1. Table showing the proportion of variance explained by each individual principal components (PCs), the cumulative proportion of variance explained by PC1+PC2 and PC1+PC2+PC3, and the contribution that each reported adverse effect has in defining each PC (i.e. PCs loadings).

	**PC1**	**PC2**	**PC3**	**PC4**	**PC5**	**PC6**	**PC7**	**PC8**	**PC9**	**PC10**	**PC11**	**PC12**	**PC13**	**PC14**	**PC15**	**PC16**	**PC17**	**PC18**	**PC19**	**PC20**	**PC21**	**PC22**	**PC23**	**PC24**	**PC25**	**PC26**	**PC27**	**PC28**
Standard deviation	0.7384	0.5051	0.4262	0.4064	0.3820	0.3696	0.3399	0.3208	0.3067	0.2827	0.2461	0.0493	0.0471	0.0404	0.0385	0.0378	0.0325	0.0287	0.0286	0.0286	0.0273	0.0243	0.0238	0.0203	0.0203	0.0202	0.0201	0.0114
Proportion of Variance	0.2871	0.1344	0.0957	0.0870	0.0768	0.0719	0.0608	0.0542	0.0495	0.0421	0.0319	0.0013	0.0012	0.0009	0.0008	0.0008	0.0006	0.0004	0.0004	0.0004	0.0004	0.0003	0.0003	0.0002	0.0002	0.0002	0.0002	0.0001
Cumulative Proportion	0.2871	0.4215	0.5172	0.6041	0.6809	0.7529	0.8137	0.8679	0.9174	0.9595	0.9914	0.9927	0.9938	0.9947	0.9955	0.9962	0.9968	0.9972	0.9976	0.9981	0.9985	0.9988	0.9991	0.9993	0.9995	0.9997	0.9999	1.0000
Gastroint	-0.003	0.001	-0.003	0.001	-0.003	0.000	-0.004	0.002	-0.005	0.002	-0.006	0.009	-0.005	-0.815	0.025	0.005	0.008	0.000	0.004	0.002	0.001	0.004	0.000	0.307	0.286	-0.120	-0.381	-0.002
Arthralgia	-0.002	0.000	-0.003	0.000	0.002	0.001	-0.001	0.000	0.002	0.003	0.002	0.006	-0.008	0.027	0.850	0.047	0.008	0.004	0.000	0.001	-0.005	0.000	0.524	-0.001	0.001	-0.002	-0.001	0.000
Asthenia	0.001	0.003	0.002	0.000	0.002	0.001	0.000	0.003	-0.001	-0.003	0.002	0.016	0.383	-0.009	0.012	0.004	-0.923	0.001	-0.005	0.003	-0.002	0.001	0.001	0.001	0.001	-0.002	-0.001	0.000
Chills	-0.301	0.219	0.054	0.133	0.649	-0.034	0.285	0.004	0.569	0.106	0.049	-0.002	-0.003	-0.006	-0.005	0.000	0.000	-0.001	0.003	-0.002	0.004	0.000	0.002	-0.001	0.001	0.000	-0.002	0.000
Photophobia	0.000	-0.001	0.001	0.001	0.000	0.000	-0.002	-0.003	-0.001	0.000	0.000	0.001	-0.001	0.002	-0.002	-0.002	-0.003	0.787	0.615	-0.054	-0.002	0.005	-0.003	-0.002	0.003	0.003	0.003	0.000
Headache	-0.288	0.006	-0.789	-0.361	-0.041	0.328	-0.096	0.165	0.099	0.071	0.058	0.004	-0.001	0.003	-0.004	-0.002	-0.002	0.002	0.000	-0.003	0.000	0.002	-0.002	0.000	0.000	0.002	0.001	-0.001
Sinus_congestion	-0.001	0.000	-0.002	0.003	0.003	0.000	0.000	-0.002	0.002	0.002	0.001	0.002	-0.005	0.015	0.523	0.029	0.004	-0.002	0.001	0.006	0.003	-0.004	-0.851	0.000	0.000	0.001	0.002	-0.001
Localized_pain	-0.429	-0.182	-0.042	0.750	-0.349	0.288	-0.029	-0.048	0.100	0.006	0.011	0.004	-0.002	0.002	-0.002	-0.001	-0.003	0.000	-0.001	0.000	0.000	0.000	0.001	0.001	0.001	0.000	0.002	0.001
Diarrhea	-0.001	0.000	0.001	0.000	-0.003	0.005	-0.003	0.004	0.004	0.003	0.002	-0.817	0.037	-0.013	0.033	-0.508	0.000	-0.006	0.007	0.000	-0.240	0.110	0.000	-0.001	-0.001	-0.001	0.003	-0.051
Inj_site_pain	0.251	-0.722	-0.294	0.235	0.464	-0.166	-0.153	-0.011	-0.070	-0.054	0.019	-0.001	0.002	-0.001	-0.002	-0.003	-0.001	0.000	-0.002	0.003	0.000	0.000	0.002	-0.001	-0.001	0.000	-0.001	0.000
Inj_site_red	0.000	0.001	-0.001	0.001	-0.001	0.003	0.001	0.001	-0.002	0.001	0.000	0.001	-0.001	0.002	-0.001	0.000	0.001	0.001	0.000	-0.002	-0.002	0.007	-0.003	-0.747	0.063	-0.533	-0.393	-0.001
Pyrexia	-0.259	0.187	-0.084	0.152	0.327	0.051	0.349	0.131	-0.784	0.039	-0.058	-0.004	0.004	0.002	0.000	0.000	0.004	0.001	0.000	0.003	-0.003	0.001	0.001	0.000	-0.002	0.003	0.003	0.000
Paresthesia	-0.001	0.003	-0.001	0.003	-0.002	0.000	-0.003	0.000	0.006	0.004	0.003	0.044	0.922	-0.003	0.005	-0.003	0.384	0.002	0.001	0.002	-0.002	-0.001	-0.001	-0.002	-0.001	0.001	0.000	0.000
Insomnia	-0.179	0.198	-0.341	0.158	-0.204	-0.861	-0.042	-0.008	0.014	0.054	0.019	-0.003	-0.001	0.003	-0.001	0.000	-0.002	0.001	0.001	0.002	-0.002	0.001	0.001	0.000	0.001	-0.002	0.000	0.000
Hypersensitivity	-0.024	0.011	-0.073	-0.002	0.018	-0.001	0.010	-0.008	0.089	-0.211	-0.970	-0.001	0.003	0.003	0.003	0.000	0.000	0.002	-0.001	-0.005	-0.005	0.004	0.000	-0.001	0.000	-0.004	0.006	0.002
Hypoesthesia	0.000	-0.001	0.001	0.001	-0.001	0.000	-0.001	0.000	0.000	0.000	0.000	-0.091	0.005	0.000	-0.013	0.270	0.002	0.001	-0.005	0.006	0.159	0.574	0.000	0.000	0.002	0.000	0.011	-0.750
Lymphadenopathy	-0.067	0.083	-0.044	0.007	0.032	-0.015	0.052	0.072	0.032	-0.964	0.219	-0.003	0.001	-0.003	0.003	0.000	0.005	0.001	-0.001	-0.003	0.000	-0.001	0.000	-0.001	0.000	0.000	-0.001	-0.001
Malaise	-0.505	-0.092	0.344	-0.167	0.173	-0.097	-0.660	0.324	-0.097	-0.003	-0.028	0.004	-0.001	0.003	0.000	0.000	0.001	0.000	-0.001	0.001	-0.003	-0.001	-0.001	-0.001	-0.001	-0.002	0.001	0.000
Nausea	-0.163	0.137	-0.067	-0.062	0.162	0.060	-0.301	-0.897	-0.134	-0.054	0.017	-0.004	0.001	0.002	-0.002	0.000	-0.002	-0.002	-0.003	-0.001	0.000	-0.001	0.001	0.002	-0.002	-0.001	-0.004	-0.001
Presyncope	0.000	0.001	0.000	0.001	-0.002	-0.001	-0.002	0.001	0.001	0.000	-0.007	0.001	-0.001	0.004	0.001	0.001	-0.002	0.001	-0.002	-0.004	-0.010	0.009	0.000	-0.253	-0.168	0.759	-0.576	-0.005
Pustola	0.000	0.001	0.001	0.000	0.001	0.000	0.001	0.000	0.003	0.001	0.000	0.000	-0.002	-0.003	-0.002	0.002	0.003	0.423	-0.522	0.219	-0.031	0.000	-0.001	0.000	-0.002	-0.001	0.001	0.000
Hyperhidrosis	-0.001	0.000	-0.002	0.001	-0.002	0.001	-0.003	0.004	-0.002	0.001	-0.006	-0.321	0.016	0.004	-0.004	0.108	0.000	0.021	-0.018	0.005	0.633	-0.638	0.006	0.000	0.002	0.002	-0.018	-0.276
Fatigue	-0.439	-0.538	0.182	-0.394	-0.185	-0.155	0.482	-0.195	0.014	-0.019	0.000	-0.003	0.004	-0.001	0.001	-0.001	-0.001	0.000	0.000	0.001	0.001	-0.001	0.000	0.000	0.000	0.000	-0.003	0.000
Sweats	0.000	-0.001	-0.001	0.001	0.001	0.001	0.002	0.000	0.003	0.001	0.001	-0.115	0.005	-0.003	-0.027	0.453	0.002	-0.018	0.024	-0.014	-0.705	-0.435	-0.002	0.000	-0.002	-0.005	0.002	-0.305
Tachycardia	0.000	-0.003	-0.001	0.000	-0.001	0.002	-0.003	0.002	0.001	0.001	0.001	-0.454	0.023	-0.001	-0.033	0.670	0.004	0.003	-0.002	0.004	0.129	0.248	0.000	0.000	0.001	0.002	-0.001	0.515
Cough	-0.001	0.002	-0.001	-0.002	-0.001	0.003	-0.001	-0.001	0.004	-0.004	-0.004	0.002	-0.003	0.004	-0.004	0.001	0.000	-0.151	0.276	0.949	-0.001	-0.007	0.006	-0.003	-0.001	0.002	-0.003	0.000
Aphtha	-0.001	0.000	-0.001	-0.001	-0.002	-0.001	-0.002	-0.002	0.000	0.001	0.001	0.004	-0.003	-0.409	0.013	0.003	0.002	-0.002	0.002	0.001	0.008	-0.008	0.002	-0.309	-0.284	0.121	0.376	0.001
Vomiting	0.000	0.001	0.001	0.000	0.001	0.000	0.001	0.000	0.003	0.001	0.000	0.000	-0.002	-0.003	-0.002	0.002	0.003	0.423	-0.522	0.219	-0.031	0.000	-0.001	0.000	-0.002	-0.001	0.001	0.000
Inj_site_swel	-0.001	0.000	-0.001	-0.001	-0.002	-0.001	-0.002	-0.002	0.000	0.001	0.001	0.004	-0.003	-0.409	0.013	0.003	0.002	-0.002	0.002	0.001	0.008	-0.008	0.002	-0.309	-0.284	0.121	0.376	0.001
Myalgia	0.000	-0.001	0.001	-0.001	0.001	-0.001	-0.002	-0.003	-0.001	0.000	0.000	0.000	0.000	0.001	0.000	0.000	0.000	-0.003	-0.005	0.001	-0.001	-0.006	0.001	-0.304	0.852	0.310	0.294	0.001

Table S2. Descriptive analysis of reported adverse effects absolute frequency across the three clusters.

	Cluster 1	Cluster 2	Cluster 3
Gastroint	3	0	0
Arthralgia	2	0	1
Asthenia	0	2	1
Chills	329	145	43
Photophobia	1	0	1
Headache	380	177	115
Sinus congestion	1	0	1
Localized pain	591	149	153
Diarrhea	2	2	1
Inj site pain	463	0	1089
Inj site red	0	1	0
Pyrexia	267	119	24
Paresthesia	1	2	2
Insomnia	241	157	89
Hypersensitivity	69	43	51
Hypoesthesia	1	0	0
Lymphadenopathy	99	81	41
Malaise	629	208	80
Nausea	183	125	31
Presyncope	0	1	0
Pustola	0	1	0
Hyperhidrosis	2	0	0
Fatigue	673	203	295
Sweats	1	0	1
Tachycardia	2	0	1
Cough	1	1	0
Aphtha	1	0	0
Vomiting	0	1	0
Inj site swel	1	0	0
Myalgia	1	0	0
