# Supplementary material for: BNT162b2 COVID-19 Vaccine Safety among Healthcare Workers of a Tertiary Hospital in Italy
Source: Vaccines (Basel). 2023 Feb 17;11(2):477. doi: 10.3390/vaccines11020477 (PMC9964542; doi:10.3390/vaccines11020477)
Supplement: Supplementary file 1 [file vaccines-11-00477-s001.zip › vaccines-2223195-supplementary/vaccines-2223195-sup-final/vaccines-2223195-supplementary materials-figures.pdf]

vaccines

MDPI

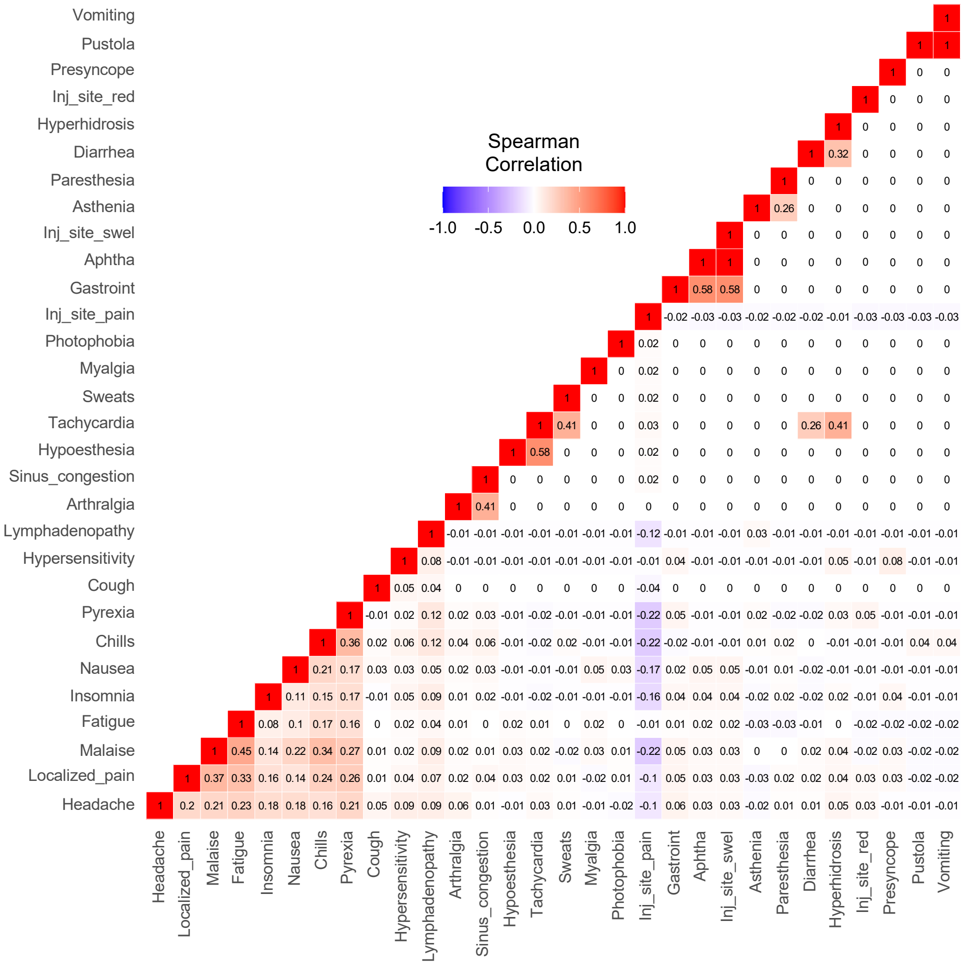

Figure S1. Spearman's correlation matrix of reported adverse reactions. Correlation coefficients (r_s) are included in cells and graphically summarized as a heatmap, with colours ranging from blue for negative values to red for positive values. There are two cases of r_s=1 (injection site swelling ~ aphtha, vomiting ~ pustola) since these pairs of adverse effects were reported only once together.

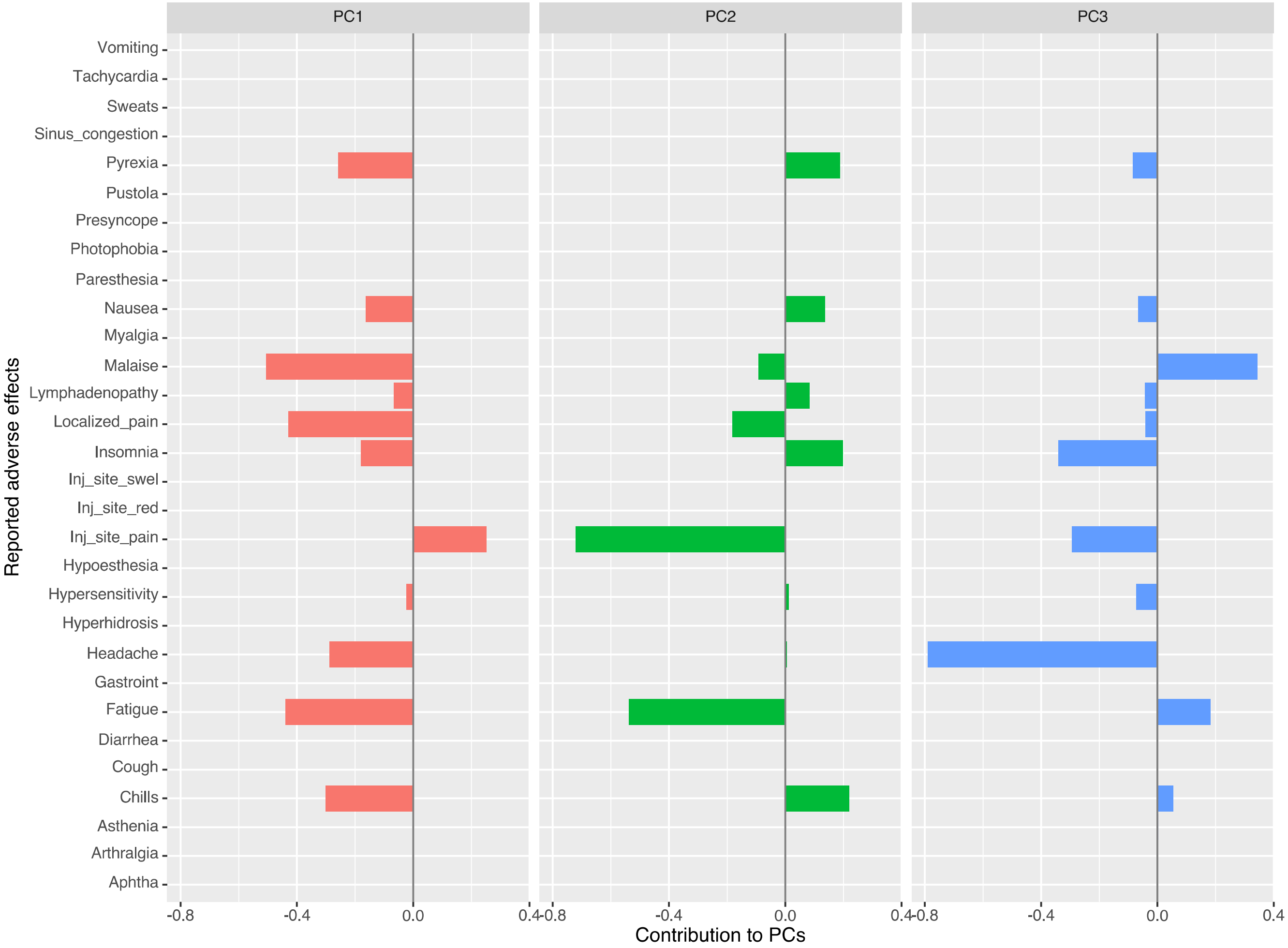

Figure S2. Bar-plot representation of the individual contribution of each reported adverse effects on the definition of the three main principal components, which together explain 51.7% of the total variation.

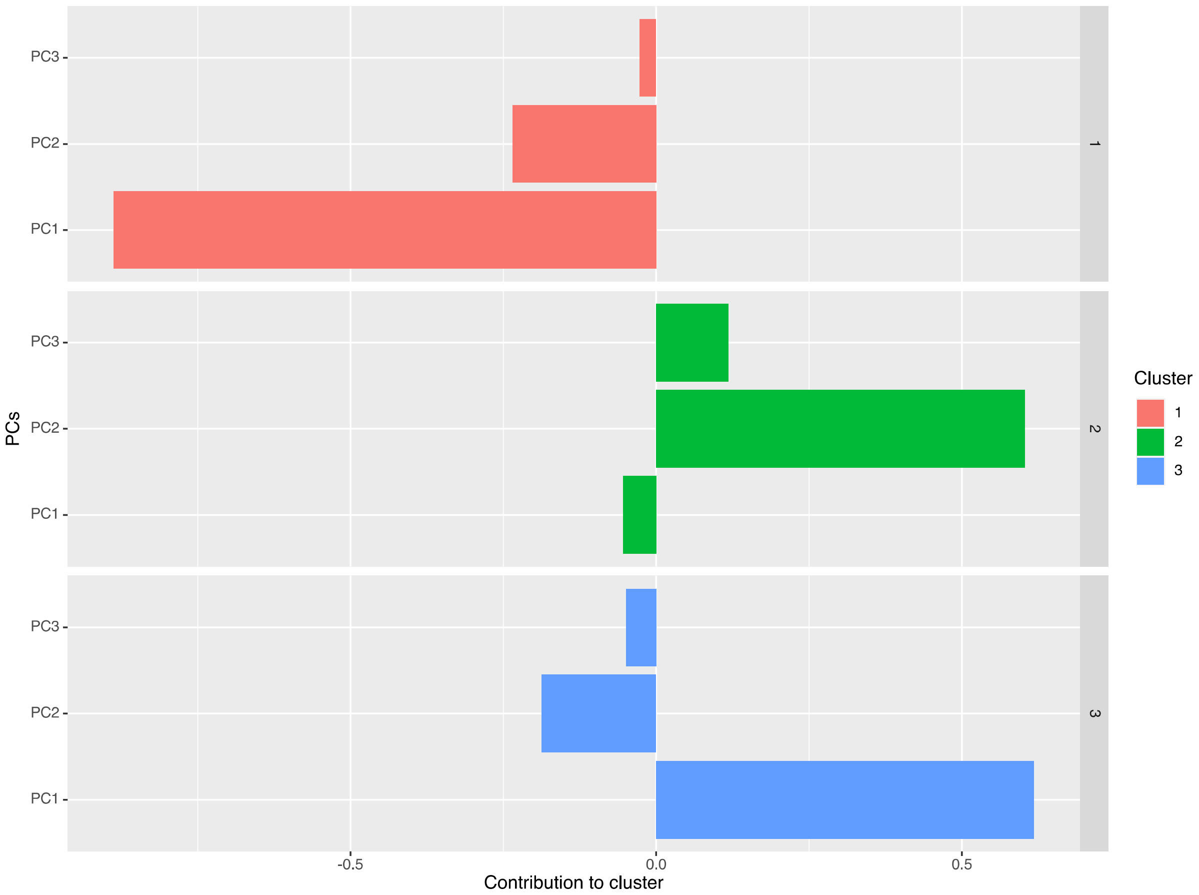

Figure S3. Bar-plot representation of the individual contribution of each identified principal component on the definition of the three clusters of reports identified through a k-means cluster analysis of principal components.
